# Effects of Pomegranate *(Punica Granatum L.)* Seed and Peel Methanolic Extracts on Oxidative Stress and Lipid Profile Changes Induced by Methotrexate in Rats

**DOI:** 10.15171/apb.2017.032

**Published:** 2017-06-30

**Authors:** Farideh Doostan, Roxana Vafafar, Parvin Zakeri-Milani, Aliasghar Pouri, Rogayeh Amini Afshar, Mehran Mesgari Abbasi

**Affiliations:** ^1^Physiology Research Center, Kerman University of Medical Sciences, Kerman, Iran.; ^2^Department of Biology, Faculty of Science, Islamic Azad University, Ahar Branch, Ahar, Iran.; ^3^Liver and Gastrointestinal Diseases Research Center, Tabriz University of Medical Sciences, Tabriz, Iran.; ^4^Faculty of Sciences, Urmia University, Urmia, Iran.; ^5^Student Research Committee, Drug Applied Research Center, Tabriz University of Medical Sciences, Tabriz, Iran.

**Keywords:** Methotrexate, Oxidative stress, Pomegranate, Rats

## Abstract

***Purpose:*** Methotrexate (MTX) is prescribed in many diseases and can result in oxidative stress (OS) followed by injuries in some tissues. Antioxidants administration are effective in reducing OS. Pomegranate exhibits high anti-oxidant capacities. This study investigated whether pomegranate seed and peel methanolic extracts (PSE and PPE) could protect against MTX-induced OS and lipid profile changes in rats.

***Methods:*** Forty-eight rats were randomly divided into 6 groups: control group (normal salin), PSE group (500 mg/kg, orally), PPE group (500 mg/kg, orally), MTX group (10 mg/kg, IM), MTX and PSE group, and MTX and PPE group. Blood samples were taken for analysis in the end of the procedure.

***Results:*** The findings showed a significant reduction in Glutathione peroxidase (GPx) and Superoxide dismutase (SOD), and an enhancement in malondialdehyde (MDA) values after MTX treatment (p < 0.05). SOD and GPx levels reached the levels of the control group in MTX+SPE and MTX+PPE groups. No significant differences were observed in catalase (CAT) and total antioxidant capacity (TAC) levels between groups. The results showed a significant decrease in total cholesterol (TC), low density lipoprotein (LDL), and high density lipoprotein (HDL) in the MTX treated group (p < 0.01). The values of TC, HDL, and LDL became elevated to the normal control levels in the MTX+PSE and MTX+PPE treated groups.

***Conclusion:*** The results showed the OS induced by MTX and the protective effects of PSE and PPE against MTX-induced serum oxidative stress and lipid profile changes in rats.

## Introduction

The presence of active oxygen species in excess of the tissue's available antioxidant buffering capacity, results in oxidative stress. Reactive oxygen species (ROS) may damage DNA, proteins, lipids, and/or carbohydrates disturbing the cells or tissues structure and function. Tissue damage and sometimes chronic human diseases may occur following enzyme and non-enzyme-mediated biochemical reactions, which produce free radicals that are extremely reactive intermediate compounds. All body tissues are exposed chronically to oxidants from endogenous and/or exogenous sources.^1,2^

Methotrexate (MTX), which has inhibitory effect on di-hydrofolatereductase, is routinely prescribed in many diseases such as cancers and autoimmune diseases. MTX suppresses DNA synthesis and adversely influences several tissues particularly the liver. The long term application of MTX causes hepatic fibrosis or cirrhosis and increases cardiovascular risk.^3,4^ MTX may affect the balance of pro-oxidants and antioxidants, which can result in the enhancement of oxidative stress, followed by injuries in some tissues. Antioxidants can be considered to reduce the OS during MTX treatment.^5,6^

Pomegranate (*Punica granatum L*.) represents a phyto-chemical reservoir that has been extensively referenced in medical folklore. This fruit has been used for centuries to treat common ailments such as microbial and parasitic infections, stomach ache, ulcers, diarrhea and dysentery. The fruit is composed of two parts: [1] the aril, that is the edible part, constitutes 52% of the total fruit (w/w), contains 78% juice and 22% seeds, and [2] the non-edible part or the peel, have been traditionally used in folk medicine. A large number of phyto-chemicals have been identified in the two parts of pomegranate, including poly-phenolics like hydrolysable tannins (ellagic and gallagic acids) and anthocyanin in the peel. The main benefit of PG has been attributed to its unique polyphenols composition, which has been shown to exhibit high anti-oxidant and anti-inflammatory capacities. The health benefits of PG consumption in preventing cardiovascular diseases and cancers have been widely investigated in both laboratory and clinical studies.^1,7-9^

This study investigated whether pomegranate seed and peel methanolic extracts (PSE and PPE) could protect against MTX-induced oxidative stress in rat blood serum.

## Materials and Methods

### Extraction

The pomegranates (*P. granatum* L.) were provided from Tabriz suburbs (East Azarbaijan, Iran). The fresh fruits (Post-Ghermez variety, 5-64-WS)^10^ were manually washed and peeled. The peels and seeds were separated and air dried in an oven (40°C, 24 h). After that, using a blender the dried materials turned into a powder. Thereafter, 500 g of pomegranate seed and pomegranate peel powders were separately extracted in methanol (Merck, Germany) (1:10 w/v) at 25°C for 24 and 96 h, respectively. The mixture of each was then filtered throw 0.45 µ pore size filters. The methanol was completely evaporated (rotary vacuum evaporator, Heidolph, Germany) at 40°C. The PSE and the PPE were stored in a deep freezer (-70°C) until use.^10^

### Animals

Forty-eight male Wistar rats, weighing 200 ± 20 g, were placed in a ventilated temperature-controlled room (22 ± 2°C) in standard cages (polycarbonate) under 12/12 h light/dark cycles. The animals were provided with clean drinking water and a standard rat diet *ad libitum*.

The animals were divided into 6 groups (n=8):

- Group I: placebo control, daily received normal saline (orally, for 18 days).
- Group II: daily received 500 mg/kg PSE (orally, for 18 days).
- Group III: daily received 500 mg/kg PPE (orally, for 18 days).
- Group IV: daily received 10 mg/kg MTX (IM, for three days beginning from the 10^th^ day).
- Group V: daily received 500 mg/kg PSE (orally, for 18 days) and also 10 mg/kg MTX (IM, for three days beginning from the 10^th^ day).
- Group VI: daily received 500 mg/kg PPE (orally, for 18 days) and also 10 mg/kg MTX (IM, for three days beginning from the 10^th^ day).

After the intervention, blood samples were obtained using cardiac puncturing method under anesthetic condition and were centrifuged at 2000 g and 4°C for 10 min. The blood serum samples were placed at a temperature of -70°C in a freezer.

### Biochemical tests 

Commercial kits (Randox, Italy) were used for determining TAC, GPX, and SOD of samples. TC, triglyceride (TG), LDL, and HDL were assayed using commercial kits (Pars Azmun, Karaj, Iran). Malondialdehyde (MDA) contents of samples were analyzed using barbituric acid method.^10^ The automated biochemistry analyzer (Alcyon 300, Abbott, USA) was used for biochemical analysis after calibration and validation. Cayman kit (USA) was used for assaying the CAT activities of the samples.

### Analyses of PSE and PPE

The antioxidant capacity of the extracts (PSE and PPE) were assayed using DPPH assay method.^10^ The quercetin RC50 (control material) was 0.004 mg/ml. Folin-Ciocalteu reagent was used for determining the total phenolic equivalent (mg of gallic acid equivalent per gram of extract, GAE/ gram extract).^11^ A spectrophotometric method was used for assaying total flavonoids.^10,12^

### Statistical Analysis

SPSS (13) for Windows (SPSS Inc., Chicago, USA) was used for statistical analyses. The normality was surveyed by using Kolmogorov–Smirnov test and Q-Q chart. For comparing between groups, we used ANOVA (One-way analysis of variance) for normally distributed data. Tukey post-hoc test was used for multiple comparisons. The data were stated as mean ± SD (standard deviation). The median ± interquartile and Wilcoxon test were used for non-normally distributed data. P-values less than 0.01 and 0.05 were statistically considered significant.

## Results and Discussion

### Analysis of PSE and PPE

Total phenolic, flavonoid compounds, and antioxidant activity of PSE and PPE were assayed and the results are shown in Table 1.

**Table 1:** Composition of pomegranate seed extract (PSE) and pomegranate peel extract (PPE).

**Sample** **(n = 3)**	**Antioxidant activity (RC50; µg/ml)**	**Total phenolic content (mg GAE/g extract)**	**Total Flavonoid (%)**
**PSE**	510.7 ± 2.5	41.1 ± 0.2	0.42 ± 0.01
**PPE**	27 ± 0.3	147.2 ± 0.2	1.17 ± 0.04

The results are expressed as means ± 1SD.

### Lipid profile contents of the samples

The TC, TG, HDL, and LDL of the blood serum samples were determined. The results are shown in Table 2. As shown in the table, MTX administration decreased TC, LDL (p<0.01), and HDL (p<0.05) levels significantly. While, the administration of SPE and MTX caused an enhancement in TG and LDL levels (p<0.01). PPE together with MTX decreased the blood serum HDL content significantly (p<0.05).

**Table 2:** Effects of pomegranate seed extract (PSE) and pomegranate peel extract (PPE) on blood serum lipid profile in rats following methotrexate (MTX) treatment.

**Parameter**	**Control**	**PSE**	**PPE**	**MTX**	**SPE + MTX**	**PPE + MTX**
**Cholesterol (mg/dl)**	97.7 ± 11.9	89.8 ± 5.6	89.6 ± 5.6	54.3 ± 13.0^**^	109.2 ± 11.8	90.7 ± 9.9
**Triglyceride (mg/dl)**	42.0 ± 6.3	42.8 ± 5.8	41.8 ± 4.1	39.7 ± 5.6	86.8 ± 14.8^**^	49.5 ± 9.9
**HDL (mg/dl)**	31.5 ± 3	27.5 ± 2.9	30.6 ± 2.5	21.0 ± 5.1^*^	31.8 ± 6.7	20.5 ± 4.3*
**LDL (mg/dl)**	37.5 ± 1.6	35.0 ± 6.7	31.4 ± 3.1	16.4 ± 4.0^**^	70.5 ± 5.2**	45.3 ± 5.2

The results are expressed as means ± 1SD. * and ** significantly different when compared with the control group (p < 0.05 and p < 0.01, respectively).

### Changes in antioxidant enzymes following MTX, PSE, and PPE administrations

**Figure 1:**
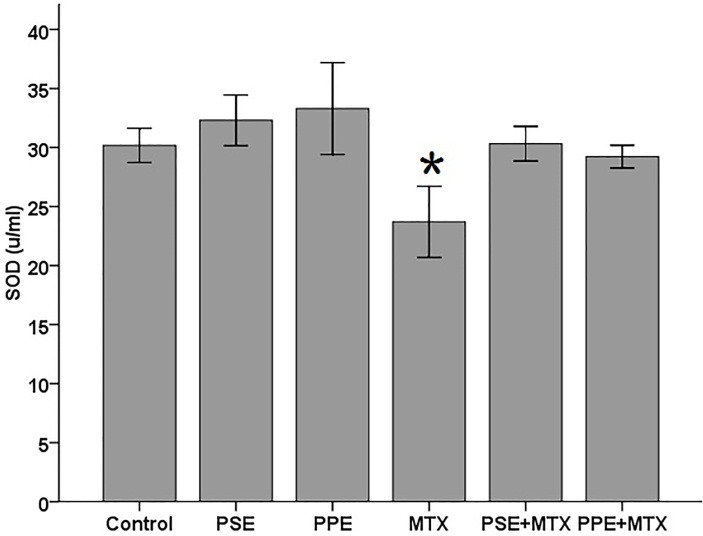
Effects of pomegranate seed extract (PSE) and pomegranate peel extract (PPE) on blood serum superoxide dismutase (SOD) content in rats following MTX treatment. The values are expressed as means ± 1SD. ^*^ significantly different at (p<0.05) when compared with the control group.

**Figure 2:**
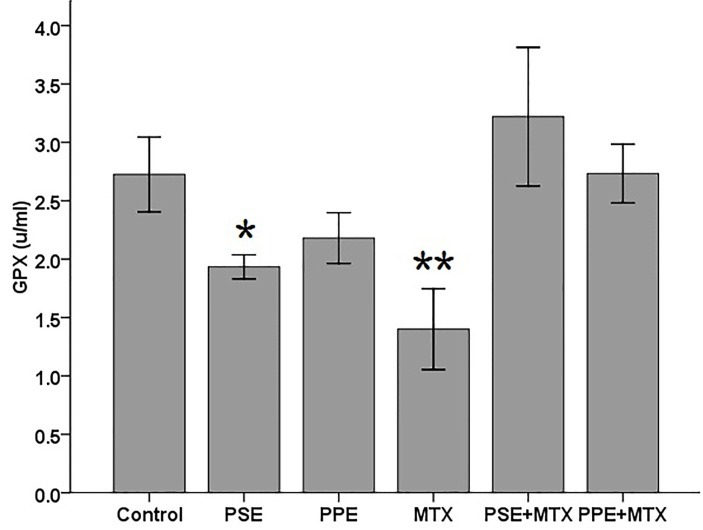
Effects of pomegranate seed extract (PSE) and pomegranate peel extract (PPE) on blood serum glutathione peroxidase (GPx) content in rats following MTX treatment. The values are expressed as means ± 1SD.^*^ and ^**^ significantly different when compared with the control group (p<0.05 and p<0.01, respectively).

**Figure 3:**
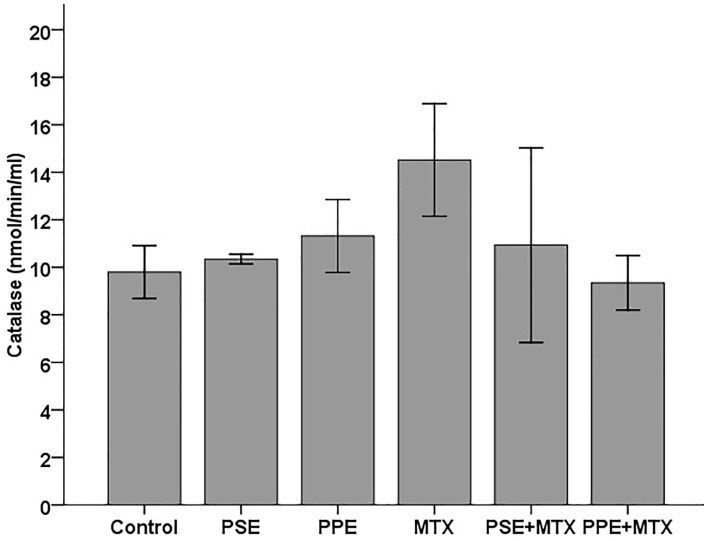
Effects of pomegranate seed extract (PSE) and pomegranate peel extract (PPE) on blood serum CAT content in rats following MTX treatment. The values are expressed as means ± 1SD.* significantly different at (p<0.05) when compared with the control group.

**Figure 4:**
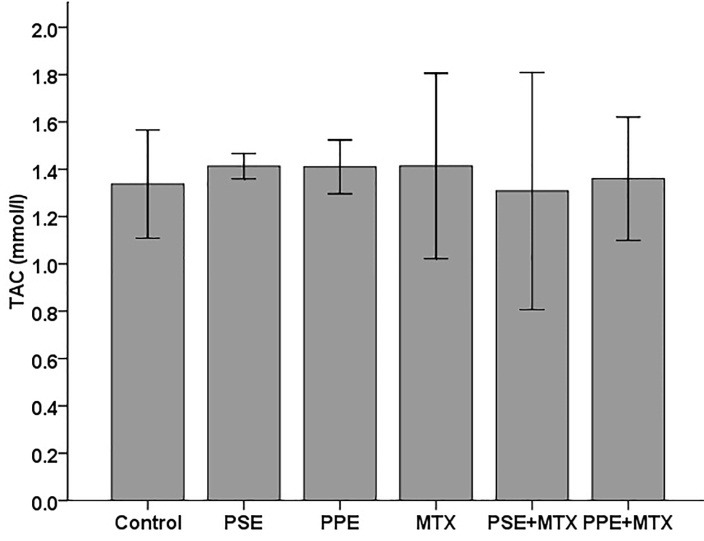
Effects of pomegranate seed extract (PSE) and pomegranate peel extract (PPE) on blood serum total antioxidant capacity (TAC) in rats following MTX treatment. The values are expressed as means ± 1SD.^*^ significantly different at (p<0.05) when compared with control group.

**Figure 5:**
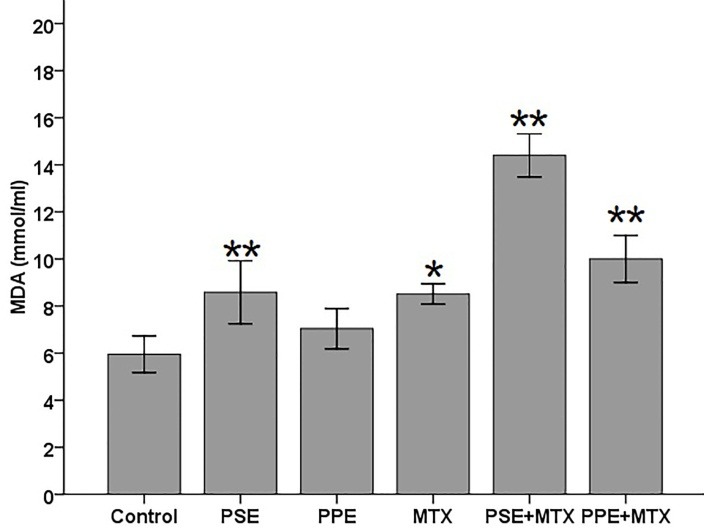
Effects of pomegranate seed extract (PSE) and pomegranate peel extract (PPE) on blood serum malondialdehyde (MDA) in rats following MTX treatment. The values are expressed as means ± 1SD.^*^ and ^**^ significantly different when compared with the control group (p<0.05 and p<0.01, respectively).

SOD, GPx, CAT, TAC, and MDA levels were assayed in the blood serum samples following MTX, PSE, and PPE administrations and the results are shown in Figures 1–5, respectively. SOD and GPx levels were significantly decreased while MDA was increased in the MTX group following MTX administration as compared with the control group (p<0.05) (Figures 1, 2, and 5). The GPx level was significantly decreased in the PSE group (Figure 2). The MDA levels showed a significant enhancement in MTX (p<0.05), PSE, MTX + PSE, and MTX + PPE (p<0.01) groups as compared with the control group (Figure 5). Other differences between MTX and extract administered groups and the control group were not statistically significant (p > 0.05).

Endogenous enzymatic and non-enzymatic antioxidants affects the unwanted effects of oxidative agents. SOD, GPx, and CAT are water-soluble antioxidants. ROS and reactive nitrogen species (RNS) may be removed by antioxidant enzymes such as SOD, GPx, and CAT. SOD is in the first anti-oxidant defense line and results in dismutation of oxygen to H_2_O_2_. GPx reduces organic peroxides including H_2_O_2_ to H_2_O and O_2_, that requires glutathione (hydrogen donor and scavenger for H_2_O_2_, hydroxyl radical and chlorinated oxidants). CAT reduces H_2_O_2_ to water.^13,14^ Evaluation of the status and the aactivity of enzymatic antioxidants, such as SOD, GPx, and CAT, can be used to assess OS. Reduction in the antioxidant defense capacity can be measured by SOD, GPx, TAC, and CAT in the serum. Decreased levels of plasma SOD, GPx, and TAC activity have been reported in some oxidative stress conditions.^13^

Malondialdehyde (MDA) is considered the important lipopolysaccharide oxidative stress marker. DNA damage and tissue injury may result in excessive MDA. MDA can react with proteins free amino-groups and form MDA-modified protein adducts.^15^ Aldehydic products, such as MDA, have relatively longer half-lives as compared with ROS. The products can diffuse to other intra- and extra-cellular places and amplify the effects of oxidative stress. ROS may damage poly-unsaturated fatty acids and cause cell organelle and membrane lipid peroxidation resulting in producing the above mentioned products.^14^

The results of this study indicated that oxidative stress was induced by MTX. The present findings showed a significant reduction in rat serum SOD (Figure 1) and GPx (Figure 2), and an enhancement in MDA values (Figure 5) after MTX treatment (p<0.05). The findings of this study also showed that the levels of SOD and GPx reached the control group levels when MTX was administered alongside PSE or PPE, suggesting the protective property of PSE and PPE against changes induced by MTX (Figures 1 and 2). There were no significant differences in CAT and TAC levels between the treated and control groups (Figure 3 and 4).

Oxidative stress induced by MTX has also been demonstrated in some previous studies. The findings of most previous investigations are in agreement with the findings of the present study. In a study by Elango *et al.*,^16^ plasma MDA was significantly increased (p<0.001) and the activities of plasma SOD, TAC, and serum CAT levels decreased (but not significant) after MTX treatment in psoriasis patients. WANG *et al.*,^7^ in their study, demonstrated that PPE and black bean peel extract, particularly a combination of both can inhibit the pancreas damage due to OS resulting in ameliorating hyperglycemia. Kumar *et al.*^17^ demonstrated that PPE administration can enhance the antioxidant defense against oxidative stress induced by mercuric chloride. In another study, SOD and GPX values were significantly higher, but TAC was significantly lower in MTX-treated animals as compared with the controls (p<0.05).^18^ Further, Shema-Didi *et al.*^19^ showed that one-year pomegranate juice (PJ) intake decreased oxidative stress and inflammation in hemodialysis patients. While Faria *et al.*^20^ demonstrated the protective effect of PJ against systemic oxidative stress in mice. In their study, SOD, GPx, and CAT activities were found to be decreased by PJ treatment.

In the present study, the administration of MTX with PSE or PPE, surprisingly, increased the serum MDA levels as compared with MTX alone and control group (Figure 5). On the other hand, PSE (but not PPE) administration significantly decreased serum GPx and increased MDA levels as compared with the control group. This indicates that PSE may induce oxidative stress alone.

On the other hand, the present findings showed a significant decrease in TC, HDL, and LDL in the MTX treated group as compared with the control group. TC, HDL, and LDL levels elevated to normal control levels and more after treatment with MTX+PSE or MTX+PPE. Unpredictably, MTX+SPE treatment caused significant enhancement in serum TG and LDL levels (p<0.01), and MTX+PPE treatment caused significant decrease in serum HDL level (p<0.05) as compared with the control group (Table 2).

The results of this study are in accordance with reports of some previous studies, but some contradict our findings regarding serum lipid profile. In line with the findings of this study, Kilic *et al.*^21^ showed that the serum concentrations of TC, HDL, and LDL decreased significantly after MTX treatment. In a study performed by Chen *et al.*,^22^ no significant differences in lipid profiles and blood lipids were observed between MTX treated and non-treated subjects. Shema-Didi *et al.*^19^ found no significant difference in TC, LDL, HDL, and triglycerides between the PJ and the placebo groups of hemodialysis patients. However, as reported by Navarro-Millán *et al.*,^23^ the TC, the mean HDL, and mean LDL levels were increased in MTX-treated rheumatoid arthritis patients as compared with the baseline, but the ratio of TC to HDL-cholesterol was decreased. Saiki *et al.*,^24^ in their study, found that TC and TG levels were elevated after MTX treatment. Some previous studies have demonstrated that pomegranate fights cardiovascular disease by different mechanisms such as reducing oxidative stress, inhibiting the oxidation of potentially harmful LDL, and quenching free radicals.^1^

Our findings of PSE and PPE analysis showed considerable antioxidant activity, total phenolic, and total flavonoid contents (Table 1). Poly-phenols are the major class of phytochemicals in pomegranate fruit and reportedly have antioxidant activity *in vivo* and *in vitro*. The antioxidant activity of dietary polyphenols include reactive species scavenging, enzyme modulation to interfere with cell signaling, and oxidative stability.^1^ PJ is a major source of soluble polyphenols such as gallic acid, ellagic acid, punicalagin and quercetin.^25^ Further, a research has demonstrated that polyphenols possess powerful antioxidant properties, which represent the most likely mechanism responsible for the protective benefits of pomegranate.^8^ The antioxidant capacity of pomegranate has been shown to be 3 times higher than that of red wine or green tea infusion.^26^ PPE is also rich in polyphenolic class antioxidants, including flavonoids like gallotannins, ellagitannins, ellagic, ferulic and gallagic acids, anthocyanins, quercetins, and catechins. The polyphenols show important biological activities including oxidation inhibition, free radical elimination, and reducing the risks of cardio-vascular diseases.^7^ It seems that ellagitannins may be responsible for the anti-mutagenic and the promising antioxidant activities of PPE. PPE exhibits strong antioxidant activities.^7^

## Conclusion

The results of this study showed the protective effects of PSE and PPE against MTX-induced serum oxidative stress (SOD and GPx) and lipid profile (TC, HDL, and LDL) changes in rats. The findings of this study also showed considerable antioxidant activity, total phenolic, and total flavonoid contents of PSE and PPE. However, further studies are needed to investigate the mechanisms of oxidative stress induction and protection, some un-expected results, and the controversies associated with previous studies.

## Acknowledgments

The authors appreciate the members of the Drug Applied Research Center and Student Research Committee of Tabriz University of Medical Sciences (Tabriz, Iran) for their instrumental and financial support.

## Ethical Issues

The animal experiments were ethically approved by Tabriz University of Medical Sciences Research Ethics Committee (code:5-4-110-60). All procedures were according to the Helsinki's humanity research declaration.

## Conflict of Interest

The authors declare no conflict of interests.
